# Seroprevalence of Brucellosis in Patients Having Complained of Joint Pain: A Case Control

**DOI:** 10.7759/cureus.41378

**Published:** 2023-07-04

**Authors:** Renu Kumari, Raj Kumar Kalyan, Amita Jain, Puneet Kumar, K K Gupta, Asmat Jahan, Yashasvi Rastogi

**Affiliations:** 1 Microbiology, King George's Medical University, Lucknow, IND; 2 Microbiology, King George's Medical College, Lucknow, IND; 3 Rheumatology, King George's Medical University, Lucknow, IND; 4 Internal Medicine, King George's Medical University, Lucknow, IND; 5 Biostatistics, University of Lucknow, Lucknow, IND

**Keywords:** enzyme linked immunosorbent assay (elisa), arthritis, elisa, joint pain, brucellosis

## Abstract

Background: Brucellosis is a neglected zoonotic disease affecting humans and animals.

Objectives: This study aimed to estimate the seroprevalence of brucellosis in patients with joint pain.

Methods: A total of 200 participants aged from 7 to 86 years were involved in this study. Blood samples were collected from all the participants for two years, from September 2019 to September 2021, and screened for *Brucella* using anti-brucella IgM ELISA and anti-brucella IgG ELISA antibodies. A questionnaire was used to collect data on socio-demographic characteristics and human brucellosis-related risk factors.

Results: Human *Brucella *seroprevalence was 19 (9.5%) for *Brucella *IgM ELISA and 23 (11.5%) for *Brucella *IgG ELISA. The sensitivity for *Brucella* IgM ELISA and *Brucella* IgG ELISA was 65.2% and 31.6%, respectively, while the specificity was 44.1% for *Brucella* IgM ELISA and 77.9% for *Brucella *IgG ELISA. All blood culture reports of all patients were negative. The principal presentation was the observable symptoms of human brucellosis: fever, headache, chills, myalgia, and Joint pain.

Conclusion: Risk factors like consumption of raw milk or their products were found to be the most important for *Brucella* infection, so the awareness or information of risk factors and the modes of transmission is much more important in control and prevention programs. General awareness about clinical symptoms should be increased, which will improve proper diagnosis and will be helpful in early treatment. An ELISA test should be considered for diagnosing brucellosis in both acute and chronic phases.

## Introduction

Brucellosis is a neglected zoonotic disease mainly induced by facultative intracellular bacteria of the genus Brucella [1]. This disease is a major ancient endemic and re-emerging disease that affects animal rearing and public health, with financial concern imputed to humans, animals, and wildlife worldwide [2]. Humans are always the accidental hosts in which using uncooked dairy products or direct contact with infected wild or unvaccinated domesticated animals via skin abrasions or mucous membranes transmits brucellosis. Antibodies for Brucella species become detectable after 1 to 2 weeks following the onset of symptoms (CDC, 2005) [3-6].

Human brucellosis, caused mainly by Brucella abortus, B. melitensis, B. suis, and B. canis, is the main cause of brucellosis in cattle, goats, sheep, pigs, and dogs. These species resist antibiotics because of their survival capability within phagocytic cells [7-10]. In humans, brucellosis is exhibited by fever and muscle and bone pain, which is unrecognized worldwide [11-13].

The global load of brucellosis in humans remains massive, with more than 500,000 new infections occurring annually worldwide, and the annual incidence varies from 2 to 500/1,000,000 populations in different geographical regions. Laboratory investigation includes serological methods such as (ELISA) enzyme-linked immunosorbent assay (anti-brucella IgM/anti-brucella IgG), blood culture, and molecular methods like conventional PCR or Real-time PCR. The management of brucellosis involves the control of the use of raw milk and milk products, the selection of biosecurity precautions in the workplace of professionals at risk of infection, and the execution of epidemiological surveillance for early detection of cases. These actions aimed to set up barriers against the modes of contamination [11-15].

The present study was conducted to determine the seroprevalence of human brucellosis amongst patients with joint pains.

## Materials and methods

Study design

Case-control study, cases were those patients who have joint pain but do not satisfy the specific criteria of particular arthritis, while controls were those patients who have joint pain with particular arthritis in which a single joint is involved, like osteoarthritis, rheumatoid, SLE, spondylitis, etc.

Inclusion & exclusion criteria

Patients presenting with unexplained arthritis with or without fever and with arthritis with a known cause, including all age groups, all indoor and outdoor patients, and patients with appropriate consent. Patients who were unwilling to give consent were excluded from the study.

Study population

The study population comprised individuals of all age groups attending the outpatient and inpatient departments of King George's Medical University, Lucknow, with a diagnosis of explained or unexplained arthritis or experiencing joint pain or illness clinically consistent with Brucella infections.

Sample size

Sample size calculation was made by using the below-mentioned formula. For calculation, seroprevalence for human brucellosis was taken from the study of Avneet Kaur et al., 2015, in their study, 100 patients' blood samples were taken for one year, and the prevalence was 6%. Because our study is for two years, we just doubled the sample size, So 200 patients' blood samples were included in this study.

n = Z2P (1-P)/d2

n=2^2^_ x0.06x94/ (0.5)^2 ^90.24 __^ ^_For one year

The sample size for one year study is 91, so the sample size for two years will be 200.

Where Z= Z score for a level of confidence [Considered 1.96 or 2 at 95% level]. P= Expected prevalence and d=Precision [Considered to be 5%]. The prevalence of brucellosis is 6%, as per the previous report of Avneet Kaur et al., 2015.

Sample collection

This case-control study was undertaken on 200 patients suffering from joint pain from September 2019 to September 2021 at King George's Medical University, Lucknow, Uttar Pradesh. Patients with joint pain between the ages of 7 to 86 were included in the study and tested for brucellosis in the department of microbiology with a 5 mL venous blood sample. The blood samples into serum were separated by centrifugation at 2000 rpm for 15 to 20 min and transferred to fresh tubes labeled accordingly. Before processing for further tests, serum samples were stored at 4°C in a freezer, and after serology, samples were stored at -80°C. Among 200 samples, only 130 patients' blood cultures were performed using the Bact/Alert automated blood culture system. All details of patients were noted, and relevant history was recorded. The main research questions were the name of the patients, age, sex, ward or departments, date of admission and date of discharge, residence, address, and occupations. In clinical data, patients were asked for fever, headache, chills, myalgia, type of arthritis, Endocarditis, and Osteoarticular complications. Laboratory investigations were included with Brucella IgM, Brucella IgG, and Blood culture. Other laboratory findings were complete blood count (CBC), liver function test (LFT), kidney function test (KFT), HIV, Hepatitis B surface antigen (HBsAg), and Hepatitis C virus (HCV). Risk factors were travel history during the last six months, Contact with animals, and History of consumption of raw milk, milk products, and animal products. Treatment was also recorded current and during the last six months. Patient consent was taken by the patients or guardians.

Serological assay

The anti-brucella IgM and IgG ELISA were performed according to manufacturer instructions using kits procured from Nova Tec Immunodiagnostic GmbH, Dietzenbach, Germany. The calculation was done by following the formula specified by the manufacturer; for this, the measured absorbance values were first converted into NovaTec Units (NTU).

**Figure 1:**
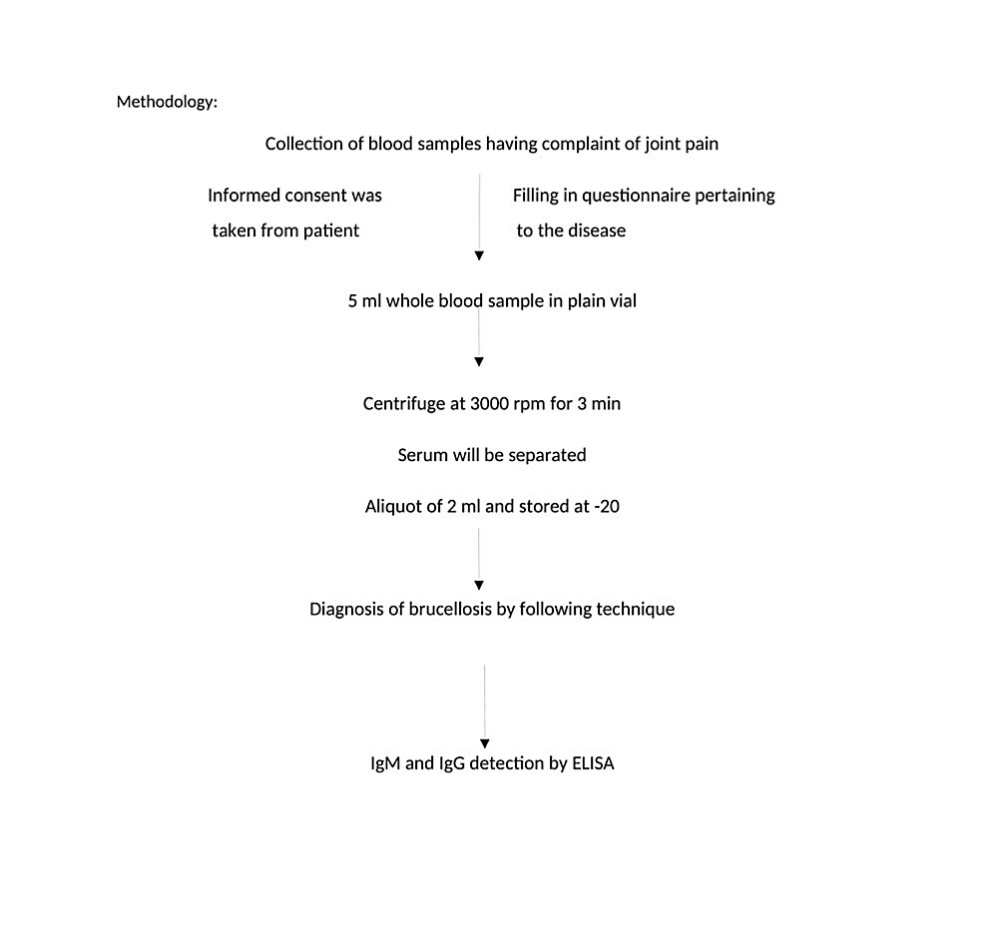
Methodology of anti-Brucella ELISA IgM and ELISA IgG.

The result was interpreted by following cut-off values > 11 NTU was considered positive, 9 NTU was considered negative, and between 9 and 11 NTU was considered equivocal as specified by the manufacturer [12,13]. Figure 1 shows the methodology of ELISA IgM/IgG for the diagnosis of antibodies against Brucella.

Blood culture

**Figure 2:**
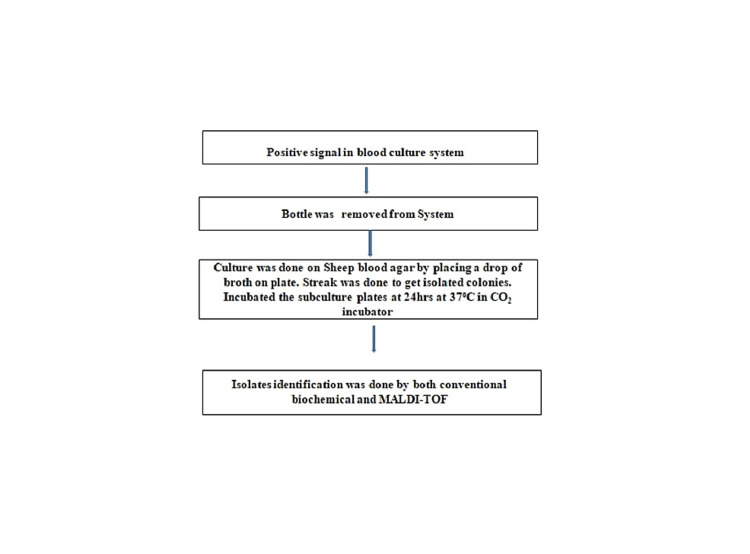
Methodology of blood culture. MALDI-TOF: matrix-assisted laser desorption ionization time-of-flight

One hundred thirty patients' blood samples were drawn and subjected to an automated blood culture system (Bact/Alert), and the cultures were incubated at 37 °C for 21 days-positive signals in the blood culture system. The bottle was removed from the system; the culture was done on Sheep blood agar by placing a drop of broth on a plate. The streak was done to get isolated colonies, and subculture plates were incubated for 24hrs at 37⁰C in the CO_2_ incubator. Isolate identification was made by conventional biochemical and matrix-assisted laser desorption ionization time-of-flight (MALDI-TOF). Figure 2 shows the methodology of blood culture using BACT/ALERT automated blood culture system.

## Results

Two hundred joint pain patients aged 7-86 years with joint pain were entered in this study. Based on the examination and clinical history, 97 (48.5%) were males (mean age = 32.38 years and S.D.=15.70), and 103 (51.5%) were females (mean age = 36.6 years and S.D.=15.71). Out of the 200 samples, 19 (9.5%) were positive for anti-brucella IgM, and 23 (11.5%) were positive for anti-brucella IgG. Of those, 1(0.5%) was positive for both anti-brucella IgM and anti-brucella IgG ELISA. The sensitivity of Brucella IgM ELISA and IgG ELISA was 65.2% and 31.6%, respectively, while the specificity was 44.1% for Brucella IgM ELISA and 77.9% for IgG ELISA, shown in Table 1.

**Table 1:** Receiver operating characteristic (ROC) curve for anti-Brucella IgM/IgG ELISA.

Characteristics	IgM	IgG
Sensitivity	65.2%	31.6%
Specificity	44.1%	77.9%
Area under curve	0.465	0.496

**Figure 3:**
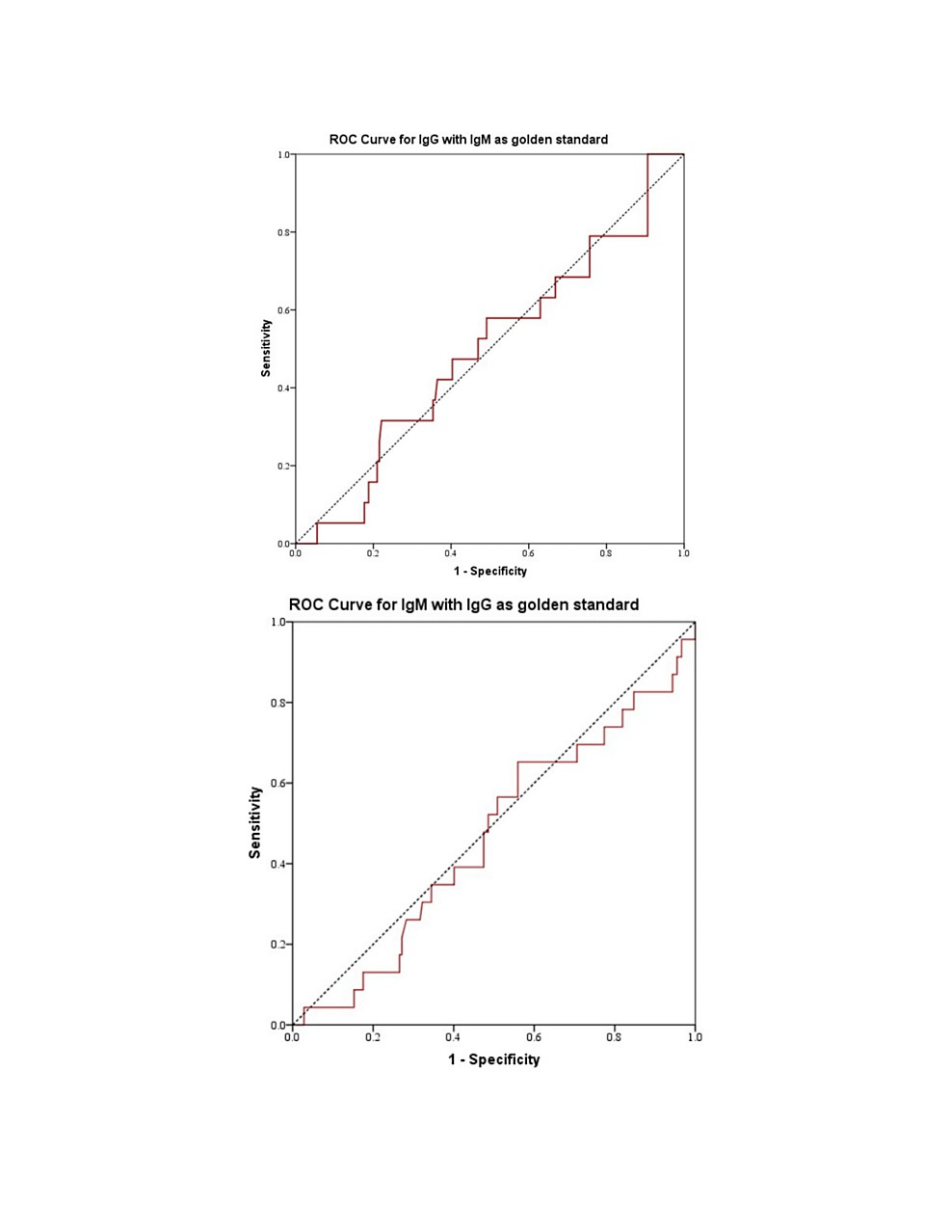
ROC analysis for IgM with IgG as a golden standard; and for IgG with IgM as a golden standard

Among the 200 patients, 161 (82%) were adults (18-60 years), 25 (12.5%) were children (18 years), and 14 (5.5%) were elderly (> 60 years). The majority of patients were from rural backgrounds; 129/200 (64.5%) and 71/200 (35.5%) belonged to urban backgrounds. Seropositive cases of 14/129 (10.8%) for anti-brucella IgM were seen in rural localities (Table 2). Analysis of cases and controls by age and sex showed that brucellosis mostly affects working-age adolescents and adult males between the ages of 7 and 69. Housewives (14/73 patients, 19.1%) and students (13/49 patients, 26.5%) were the major occupational groups seen affected in the current study.

**Table 2:** Demographical association with Brucella seroprevalence in case and control patients:

Demographic factors	Anti brucella IgM (n = 200)				Anti brucella IgG (n = 200)			
	Case (n = 5)		Control (n = 14)		Case (n = 11)		Control (n = 12)	
	N (%)	Chi-square statistic (p-value)	N (%)	Chi-square statistic (p-value)	N (%)	Chi-square statistic (p-value)	N (%)	Chi-square statistic (p-value)
Age								
Children	0 (0.0)	0.511 (0.475)	1 (1.0)	0.452 (0.501)	4 (4.0)	5.354 (0.021)	3 (3.0)	2.954 (0.086)
Adults	5 (5.0)		12 (12.0)		6 (6.0)		9 (9.0)	
Elder	0 (0.0)		1 (1.0)		1 (1.0)		0 (0.0)	
Sex								
Male	2 (2.0)	2.404 (0.304)	6 (6.0)	1.002 (0.711)	8 (8.0)	3.669 (0.157)	6 (6.0)	3.609 (0.139)
Female	3 (3.0)		8 (8.0)		3 (3.0)		6 (6.0)	
Residence								
Rural	4 (4.0)	0.526 (0.846)	10 (10.0)	1.039 (0.723)	7 (7.0)	1.158 (0.651)	6 (6.0)	1.233 (0.632)
Urban	1 (1.0)		4 (4.0)		4 (4.0)		6 (6.0)	
Occupation								
Business	0 (0.0)	9.859 (0.934)	1 (1.0)	17.331 (0.569)	0 (0.0)	14.203 (0.319)	0 (0.0)	11.910 (0.578)
Farmer	0 (0.0)		1 (1.0)		1 (1.0)		0 (0.0)	
House wife	3 (3.0)		7 (7.0)		0 (0.0)		4 (4.0)	
Medical Staff	0 (0.0)		0 (0.0)		0 (0.0)		0 (0.0)	
Student	1 (1.0)		2 (2.0)		4 (4.0)		5 (5.0)	
Teacher	0 (0.0)		1 (1.0)		2 (2.0)		1 (1.0)	
Unemployed	1 (1.0)		1 (1.0)		3 (3.0)		1 (1.0)	
Others	0 (0.0)		1 (1.0)		1 (1.0)		1 (1.0)	

Contacts or associations with animals (buffalo, cow, dog, etc.) were noticed in 7 (36.8%) for anti-brucella IgM and 8 (34.7%) for anti-brucella IgG in patients, and consumption of animal products was noticed in 8 (42.1%) for anti-brucella IgM and 8 (34.7%) for anti-brucella IgG patients (Table 3).

**Table 3:** Risk factors association with Brucella seroprevalence in case and control patients:

Risk factors	Anti brucella IgM (n = 200)				Anti brucella IgG (n = 200)			
	Case (n = 5)		Control (n = 14)		Case (n = 11)		Control (n = 12)	
	N (%)	Chi-square statistic (p-value)	N (%)	Chi-square statistic (p-value)	N (%)	Chi-square statistic (p-value)	N (%)	Chi-square statistic (p-value)
Travel history								
Yes	1 (1.0)	0.454 (1.000)	0 (0.0)	1.689 (1.000)	3 (3.0)	0.806 (0.784)	0 (0.0)	0.451 (1.000)
No	4 (4.0)		14 (14.0)		8 (8.0)		12 (12.0)	
Contact with animal								
Yes	3 (3.0)	1.536 (0.532)	4 (4.0)	1.982 (0.477)	6 (6.0)	0.791 (0.795)	2 (2.0)	2.994 (0.200)
No	2 (2.0)		10 (10.0)		5 (5.0)		10 (10.0)	
Animal product consumption								
Yes	3 (3.0)	0.745 (0.862)	5 (5.0)	12.524 (0.001)	7 (7.0)	2.171 (0.339)	1 (1.0)	0.294 (1.000)
No	2 (2.0)		9 (9.0)		4 (4.0)		11 (11.0)	

The observable symptoms of human brucellosis are fever, headache, chills, and myalgia. Joint pain was the principal presentation. Examining the clinical signs, all patients manifested joint pain (100%), followed by fever, headache, chills, and myalgia, the other common signs or symptoms in this study. There was a significant correlation between clinical signs such as night sweats (p = 0.032) and myalgia (p = 0.036) with Brucella seropositivity for anti-brucella IgM in the control group. However, headache (p = 0.005)) is a significant factor associated with Brucella seropositivity for anti-brucella IgM in the case group of patients. The statistical association of the clinical features of seropositive cases or controls is shown in Table 4.

**Table 4:** Clinical features and association with Brucella seropositivity in case and control patients:

Clinical features	Anti brucella IgM (n = 200)				Anti brucella IgG (n = 200)			
	Case (n = 5)		Control (n = 14)		Case (n = 11)		Control (n = 12)	
	N (%)	Chi-square statistic (p-value)	N (%)	Chi-square statistic (p-value)	N (%)	Chi-square statistic (p-value)	N (%)	Chi-square statistic (p-value)
Fever								
Yes	2 (2.0)	2.325 (0.335)	3 (3.0)	4.937 (0.092)	10 (10.0)	6.470 (0.031)	1 (1.0)	0.690 (0.736)
No	3 (3.0)		11 (11.0)		1 (1.0)		11 (11.0)	
Headache								
Yes	5 (5.0)	7.819 (0.005)	2 (2.0)	5.448 (0.076)	3 (3.0)	2.084 (0.370)	0 (0.0)	1.974 (0.296)
No	0 (0.0)		12 (12.0)		8 (8.0)		12 (12.0)	
Chills								
Yes	3 (3.0)	2.143 (0.412)	2 (2.0)	3.332 (0.248)	3 (3.0)	0.637 (0.836)	0 (0.0)	2.378 (0.391)
No	2 (2.0)		12 (12.0)		8 (8.0)		12 (12.0)	
Night Sweat								
Yes	0 (0.0)	0.272 (1.000)	0 (0.0)	8.548 (0.032)	0 (0.0)	3.525 (0.131)	0 (0.0)	3.994 (0.179)
No	5 (5.0)		14 (14.0)		11 (11.0)		12 (12.0)	
Myalgia								
Yes	2 (2.0)	0.323 (1.000)	1 (1.0)	7.076 (0.036)	3 (3.0)	1.685 (0.451)	0 (0.0)	0.467 (1.000)
No	3 (3.0)		13 (13.0)		8 (8.0)		12 (12.0)	
Arthralgia								
Yes	5 (5.0)	-	0 (0.0)	-	11 (11.0)	-	0 (0.0)	-
No	0 (0.0)		14 (14.0)		0 (0.0)		12 (12.0)	

## Discussion

Brucellosis is considered endemic in all states of India. The prevalence or proportion of animal brucellosis is well recognized, but human brucellosis is neglected, underestimated, and summarized. Although several cases have been reported from different parts or regions of the country, the true burden of the disease remains unrecognized [16-18]. However, a study in southern India revealed the highest brucellosis prevalence of 96.8% from serology and 70.2% from culture [17]. Similarly, brucellosis prevalence has been widely reported in different regions or parts of India. In the Goa region, 4.25%, 3.54%, 6.02%, and 4.96% of samples were positive by Rose Bengal Plate test (RBPT), serum agglutination test (SAT), indirect ELISA, and anti-brucella IgG ELISA, respectively. In a study in the Junagadh region of Gujarat, Avneet Kaur et al. reported an overall prevalence of RBPT and STAT of 9.3% and 5.3% in human samples and 7.9% and 7% in animals, respectively [21]fs. H. K. Sharma et al. (2016) reported an overall seroprevalence of brucellosis of 4.96% in the district of Jammu. Vijay Sharma et al., 1.33% was recorded in areas of the Jammu region [19-23].

The prevalence of human brucellosis was remarkably greater in rural areas compared to urban areas. This can be associated with increased human-animal connection or close contact in rural areas. Instead, Avneet Kaur et al. reported a higher prevalence of human brucellosis in rural areas than in urban areas, although the differences were not statistically significant [21]. There is no sex-wise distinction between brucellosis infection or contamination; males and females are uniformly susceptible if exposure occurs to potential risk factors. In the present study, being domiciled in rural areas, lack of knowledge about zoonotic diseases, contact with unvaccinated domestic or wild animals, raising animals, occupation-related issues, and eating during working hours at a working place was identified as the major risk factors [24].

The present study aims to estimate the seroprevalence of brucellosis in humans attending a tertiary care hospital, King George's Medical University, Lucknow, U.P. The overall prevalence recorded in this study was 9.5% and 11.5% by anti-brucella IgM and IgG ELISA, and the study concluded that brucellosis is more frequent in explained arthritis (control) patients than unexplained arthritis (cases) patients. Brucellosis is more common in the control group, meaning brucellosis also plays a role in causing arthritis. Diagnostic yields of ELISA are depicted in Table 5. This study's limitations were that we could not do blood cultures of all samples; only 130 patients' blood samples were collected. The rest of the patients did not agree to give their samples for culture tests because they were outdoor patients. Moreover, we were not so sick. Cross-reactivity was also not ruled out for anti-brucella IgM antibodies.

**Table 5:** Sero-prevalence of Brucella in case and control patients by anti-Brucella IgM and anti-Brucella IgG ELISA.

Tests	Case	Control	Chi-square statistic	p-value
	N (%)	N (%)		
Anti brucella IgM				
Negative	92 (46.0)	85 (42.5)	5.432	0.059
Equivocal	3 (1.5)	1 (0.5)		
Positive	5 (2.5)	14 (7.0)		
Anti brucella IgG				
Negative	82 (41.0)	83 (41.5)	0.383	0.920
Equivocal	7 (3.5)	5 (2.5)		
Positive	11 (5.5)	12 (6.0)		

## Conclusions

Our study concluded that human brucellosis circulates or exists in Uttar Pradesh District. Consumption of raw milk or their products was found to be the most important risk factor for Brucella infection. Thus, the awareness or information of risk factors and the modes of transmission is much more important in control and prevention programs. An extensive public awareness campaign and strict and mandatory animal movement control are needed to rein in this disease. Extension education campaigns are needed to raise awareness of the risk to veterinarians and animal owners. Besides, regular disease monitoring needs to be undertaken at the local and national levels.
